# Supplementary material for: TCGA-Reports: A machine-readable pathology report resource for benchmarking text-based AI models
Source: Patterns (N Y). 2024 Feb 21;5(3):100933. doi: 10.1016/j.patter.2024.100933 (PMC10935496; doi:10.1016/j.patter.2024.100933)
Supplement: Document S1. Figures S1‒S5 and Tables S1 and S2 [file mmc1.pdf]

Patterns, Volume *5*

Supplemental information

TCGA-Reports: A machine-readable pathology report resource for benchmarking text-based AI models

Jenna Kefeli and Nicholas Tatonetti

Figure S1. Related to Figure 1 and Methods ("Text Extraction and OCR Post-Processing").
Report Examples – TCGA-Inserted Within-Report Metadata Artifacts.
(A) Redaction Bars (B) TCGA Barcode (C) TCGA QC Table and Handwritten Annotation.

(A)

Source of Specimen:

A. Whipple
B. Left tube, ovary, uterus, and right adnexal mass
C. Appendix
D. Gallbladder
E. Ovary and fallopian tube;right

Intraoperative Diagnosis:

A. Whipple procedure: Pancreatic mass, pelvic mass: FSA1) pancreatic margin negative. FSA2) Bile duct margin negative. [redacted] The intraoperative interpretation(s) was/were performed and rendered at [redacted]

B. FS: Left tube, ovary, uterus and right adnexal mass: Favor leiomyoma. [redacted] The intraoperative interpretation(s) was/were performed and rendered at [redacted]

(B)

Surgery date: , Surgical Pathology

DIAGNOSIS:

D. Head of pancreas, duodenum, and common bile duct; Whipple resection: Invasive grade 3 (of 4) adenocarcinoma with mucinous features is identified forming a diffusely infiltrative mass (4.5 x 2.8 x 2.2 cm) in the region of the pancreatic head and possibly arising from an intraductal papillary mucinous neoplasm (2.0 x 1.5 x 1.5 cm). Tumor extends beyond the pancreas to involve peripancreatic soft tissue and the adjacent duodenal wall. The stomach and gallbladder are uninvolved by tumor. Angiolymphatic invasion is identified. Tumor involves the common bile duct and pancreatic duct. The uncinate margin is involved by tumor. The portal vein groove is negative for tumor. The pancreatic body and common hepatic duct margins are negative for tumor (see parts B and C below); however, the uncinate margin remains positive for tumor. Multiple (7 of 21) peripancreatic lymph nodes are positive for metastatic carcinoma.

- Per TSS - 0% mucinous.

UUID:224801AA-21C4-4070-B1FE-BAE31CC38318
TCGA-2J-AAB1-01A-PR Redacted

B. Pancreas, body margin, excision: Negative for tumor.
C. Common hepatic duct, margin, excision: Negative for tumor.

(C)

[illegible]
tumor is Gleason 5+5

Criteria 1/9/14	Yes	No
Diagnosis Discrepancy		✓
Primary Tumor Site Discrepancy		✓
HIPAA Discrepancy		✓
Prior Malignancy History		✓
Dual/Synchronous Primary Noted		✓
Case is (circle): (QUALIFIED) / DISQUALIFIED		
Reviewer Initials [illegible] Date Reviewed: 12/30/2013		

Figure S2. Related to Methods ("Form Detection and Removal"). **Report Examples – Removed Forms.** (A) TCGA Missing Pathology Report Form (B) TCGA Pathologic Diagnosis Discrepancy Form (C) Additional Multiple-Choice Forms, demonstrating variability.

(A)

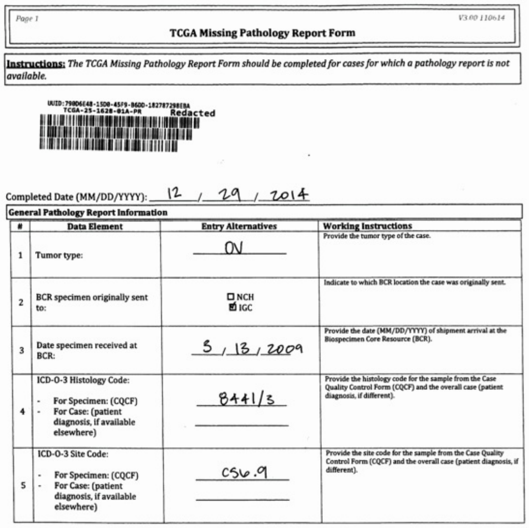

Page 1 — V3.00 110614

TCGA Missing Pathology Report Form

Instructions: The TCGA Missing Pathology Report Form should be completed for cases for which a pathology report is not available.

Redacted

Completed Date (MM/DD/YYYY): 12 / 29 / 2014

General Pathology Report Information

#	Data Element	Entry Alternatives	Working Instructions
1	Tumor type:	OV	Provide the tumor type of the case.
2	BCR specimen originally sent to:	☐ NCH ☒ IGC	Indicate to which BCR location the case was originally sent.
3	Date specimen received at BCR:	5 / 13 / 2009	Provide the date (MM/DD/YYYY) of shipment arrival at the Biospecimen Core Resource (BCR).
4	ICD-O-3 Histology Code: - For Specimen: (CQCF) - For Case: (patient diagnosis, if available elsewhere)	8441/3	Provide the histology code for the sample from the Case Quality Control Form (CQCF) and the overall case (patient diagnosis, if different).
5	ICD-O-3 Site Code: - For Specimen: (CQCF) - For Case: (patient diagnosis, if available elsewhere)	C56.9	Provide the site code for the sample from the Case Quality Control Form (CQCF) and the overall case (patient diagnosis, if different).

(B)

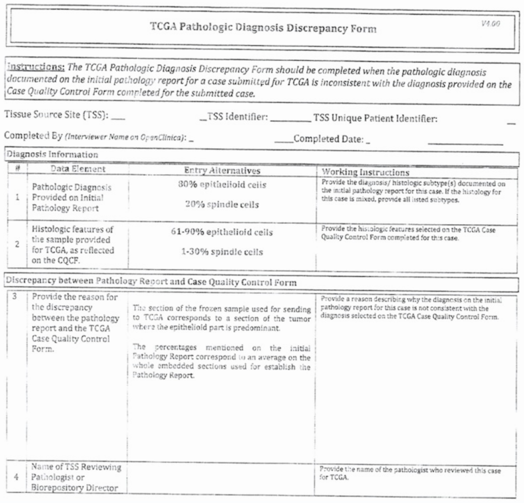

TCGA Pathologic Diagnosis Discrepancy Form — V1.00

Instructions: The TCGA Pathologic Diagnosis Discrepancy Form should be completed when the pathologic diagnosis documented on the initial pathology report for a case submitted for TCGA is inconsistent with the diagnosis provided on the Case Quality Control Form completed for the submitted case.

Tissue Source Site (TSS): ___ _TSS Identifier: ______ TSS Unique Patient Identifier: _

Completed By (Interviewer Name on OpenClinica): _ ____Completed Date: _

Diagnosis Information

#	Data Element	Entry Alternatives	Working Instructions
1	Pathologic Diagnosis Provided on Initial Pathology Report	80% epithelioid cells; 20% spindle cells	Provide the diagnosis/ histologic subtype(s) documented on the initial pathology report for this case. If the histology for this case is mixed, provide all listed subtypes.
2	Histologic features of the sample provided for TCGA, as reflected on the CQCF.	61-90% epithelioid cells; 1-30% spindle cells	Provide the histologic features selected on the TCGA Case Quality Control Form completed for this case.

Discrepancy between Pathology Report and Case Quality Control Form

#	Data Element	Entry Alternatives	Working Instructions
3	Provide the reason for the discrepancy between the pathology report and the TCGA Case Quality Control Form.	The section of the frozen sample used for sending to TCGA corresponds to a section of the tumor where the epithelioid part is predominant. The percentages mentioned on the initial Pathology Report correspond to an average on the whole embedded sections used for establish the Pathology Report.	Provide a reason describing why the diagnosis on the initial pathology report for this case is not consistent with the diagnosis selected on the TCGA Case Quality Control Form.
4	Name of TSS Reviewing Pathologist or Biorepository Director		Provide the name of the pathologist who reviewed this case for TCGA.

(C)

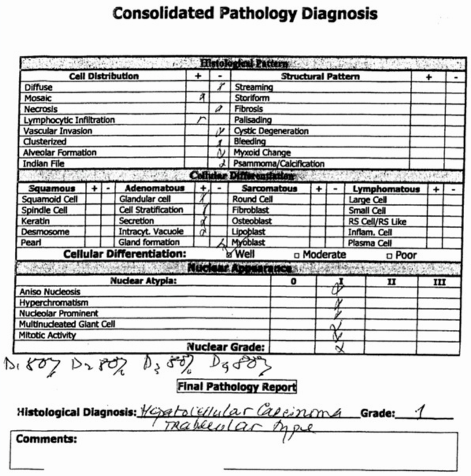

Consolidated Pathology Diagnosis

Histological Pattern; Cell Distribution: Diffuse, Mosaic, Necrosis, Lymphocytic Infiltration, Vascular Invasion, Clusterized, Alveolar Formation, Indian File; Structural Pattern: Streaming, Storiform, Fibrosis, Palisading, Cystic Degeneration, Bleeding, Myxoid Change, Psammoma/Calcification

Cellular Differentiation: Squamous (Squamoid Cell, Spindle Cell, Keratin, Desmosome, Pearl); Adenomatous (Glandular cell, Cell Stratification, Secretion, Intracyt. Vacuole, Gland formation); Sarcomatous (Round Cell, Fibroblast, Osteoblast, Lipoblast, Myoblast); Lymphomatous (Large Cell, Small Cell, RS Cell/RS Like, Inflam. Cell, Plasma Cell)

Cellular Differentiation: ☒ Well ☐ Moderate ☐ Poor

Nuclear Appearance — Nuclear Atypia: 0 / I / II / III: Aniso Nucleosis, Hyperchromatism, Nucleolar Prominent, Multinucleated Giant Cell, Mitotic Activity

Nuclear Grade:

D1 50% D2 80% D3 80% D4 80%

Final Pathology Report

Histological Diagnosis: Hepatocellular Carcinoma Trabecular type Grade: 1

Comments:

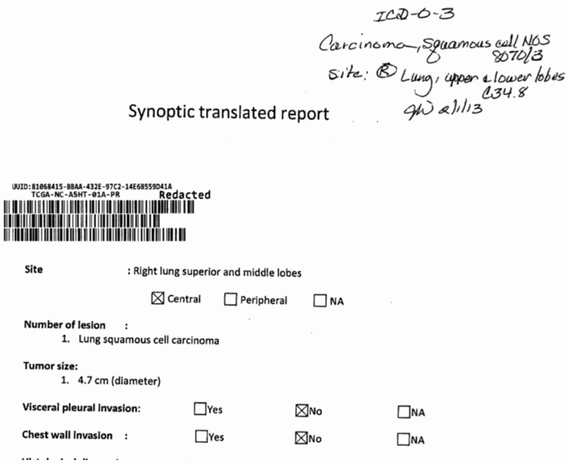

ICD-O-3

Carcinoma, Squamous cell NOS 8070/3

Site: Ⓡ Lung, upper & lower lobes C34.8

GW 2/1/13

Synoptic translated report

Redacted

Site : Right lung superior and middle lobes

☒ Central ☐ Peripheral ☐ NA

Number of lesion :

1. Lung squamous cell carcinoma

Tumor size:

1. 4.7 cm (diameter)

Visceral pleural invasion: ☐ Yes ☒ No ☐ NA

Chest wall invasion : ☐ Yes ☒ No ☐ NA

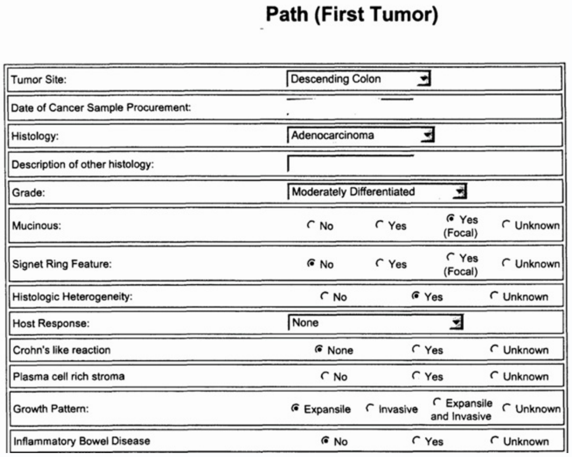

Path (First Tumor)

Tumor Site:	Descending Colon
Date of Cancer Sample Procurement:	
Histology:	Adenocarcinoma
Description of other histology:	
Grade:	Moderately Differentiated
Mucinous:	○ No ○ Yes ● Yes (Focal) ○ Unknown
Signet Ring Feature:	● No ○ Yes ○ Yes (Focal) ○ Unknown
Histologic Heterogeneity:	○ No ● Yes ○ Unknown
Host Response:	None
Crohn's like reaction	● None ○ Yes ○ Unknown
Plasma cell rich stroma	○ No ○ Yes ○ Unknown
Growth Pattern:	● Expansile ○ Invasive ○ Expansile and Invasive ○ Unknown
Inflammatory Bowel Disease	● No ○ Yes ○ Unknown

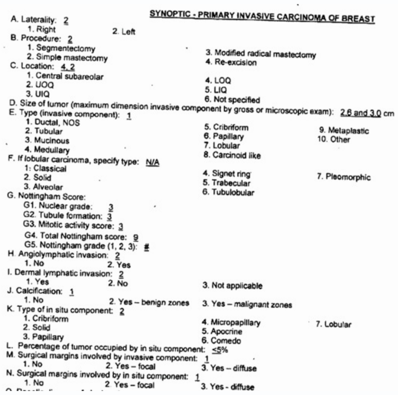

SYNOPTIC - PRIMARY INVASIVE CARCINOMA OF BREAST

A. Laterality: 2
1. Right 2. Left
B. Procedure: 2
1. Segmentectomy 2. Simple mastectomy 3. Modified radical mastectomy 4. Re-excision
C. Location: 4, 2
1. Central subareolar 2. UOQ 3. UIQ 4. LOQ 5. LIQ 6. Not specified
D. Size of tumor (maximum dimension invasive component by gross or microscopic exam): 2.6 and 3.0 cm
E. Type (invasive component): 1
1. Ductal, NOS 2. Tubular 3. Mucinous 4. Medullary 5. Cribriform 6. Papillary 7. Lobular 8. Carcinoid like 9. Metaplastic 10. Other
F. If lobular carcinoma, specify type: N/A
1. Classical 2. Solid 3. Alveolar 4. Signet ring 5. Trabecular 6. Tubulolobular 7. Pleomorphic
G. Nottingham Score:
G1. Nuclear grade: 3
G2. Tubule formation: 3
G3. Mitotic activity score: 3
G4. Total Nottingham score: 9
G5. Nottingham grade (1, 2, 3): #
H. Angiolymphatic invasion: 2
1. No 2. Yes
I. Dermal lymphatic invasion: 2
1. Yes 2. No 3. Not applicable
J. Calcification: 1
1. No 2. Yes – benign zones 3. Yes – malignant zones
K. Type of in situ component: 2
1. Cribriform 2. Solid 3. Papillary 4. Micropapillary 5. Apocrine 6. Comedo 7. Lobular
L. Percentage of tumor occupied by in situ component: ≤5%
M. Surgical margins involved by invasive component: 1
1. No 2. Yes – focal 3. Yes – diffuse
N. Surgical margins involved by in situ component: 1
1. No 2. Yes – focal 3. Yes - diffuse

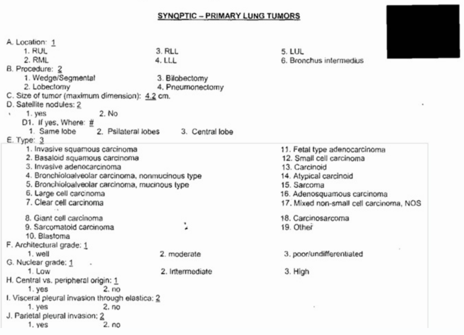

SYNOPTIC – PRIMARY LUNG TUMORS [redacted]

A. Location: 1
1. RUL 2. RML 3. RLL 4. LLL 5. LUL 6. Bronchus intermedius
B. Procedure: 2
1. Wedge/Segmental 2. Lobectomy 3. Bilobectomy 4. Pneumonectomy
C. Size of tumor (maximum dimension): 4.2 cm.
D. Satellite nodules: 2
1. yes 2. No
D1. If yes, Where: #
1. Same lobe 2. Psilateral lobes 3. Central lobe
E. Type: 3
1. Invasive squamous carcinoma 2. Basaloid squamous carcinoma 3. Invasive adenocarcinoma 4. Bronchioloalveolar carcinoma, nonmucinous type 5. Bronchioloalveolar carcinoma, mucinous type 6. Large cell carcinoma 7. Clear cell carcinoma 8. Giant cell carcinoma 9. Sarcomatoid carcinoma 10. Blastoma 11. Fetal type adenocarcinoma 12. Small cell carcinoma 13. Carcinoid 14. Atypical carcinoid 15. Sarcoma 16. Adenosquamous carcinoma 17. Mixed non-small cell carcinoma, NOS 18. Carcinosarcoma 19. Other
F. Architectural grade: 1
1. well 2. moderate 3. poor/undifferentiated
G. Nuclear grade: 1
1. Low 2. Intermediate 3. High
H. Central vs. peripheral origin: 1
1. yes 2. no
I. Visceral pleural invasion through elastica: 2
1. yes 2. no
J. Parietal pleural invasion: 2
1. yes 2. no

Figure S3. Related to Figure 1 and Methods (“Table Detection and Removal”).
TCGA QC Table – Automated Max Bounding Box Detection Example.

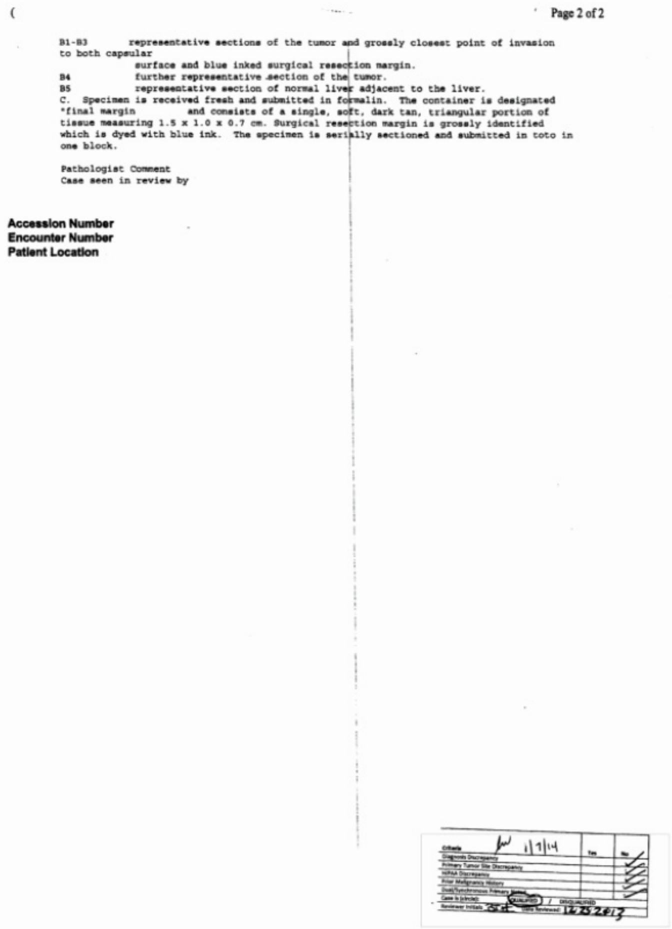

Page 2 of 2

B1-B3 representative sections of the tumor and grossly closest point of invasion to both capsular
surface and blue inked surgical resection margin.
B4 further representative section of the tumor.
B5 representative section of normal liver adjacent to the liver.
C. Specimen is received fresh and submitted in formalin. The container is designated "final margin and consists of a single, soft, dark tan, triangular portion of tissue measuring 1.5 x 1.0 x 0.7 cm. Surgical resection margin is grossly identified which is dyed with blue ink. The specimen is serially sectioned and submitted in toto in one block.

Pathologist Comment
Case seen in review by

Accession Number
Encounter Number
Patient Location

Criteria	Yes	No
Diagnosis Discrepancy		✓
Primary Tumor Site Discrepancy		✓
HIPAA Discrepancy		✓
Prior Malignancy History		✓
Dual/Synchronous Primary Noted		✓
Case is (circle): QUALIFIED / DISQUALIFIED		
Reviewer Initials: [illegible] Date Reviewed: [illegible]		

Figure S4. Related to Figure 3. **Average ROC (A) and PR (B) curves for all tissue models** (10 epochs, 10 random seeds, test set performance). Plots are ordered according to descending prevalence within the final post-processed dataset.

(A)

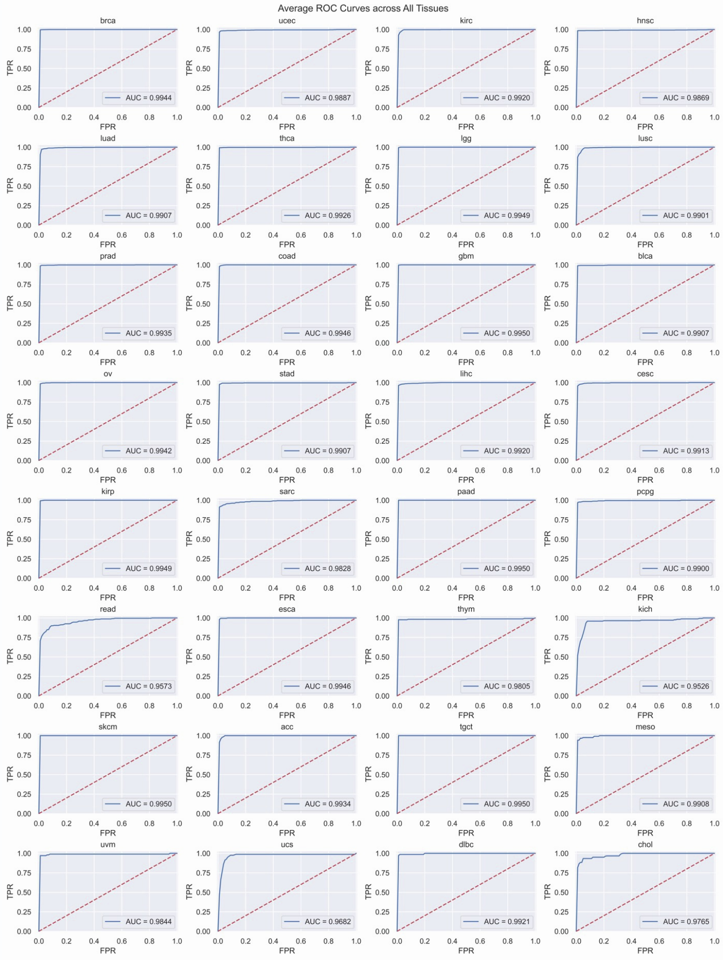

(B)

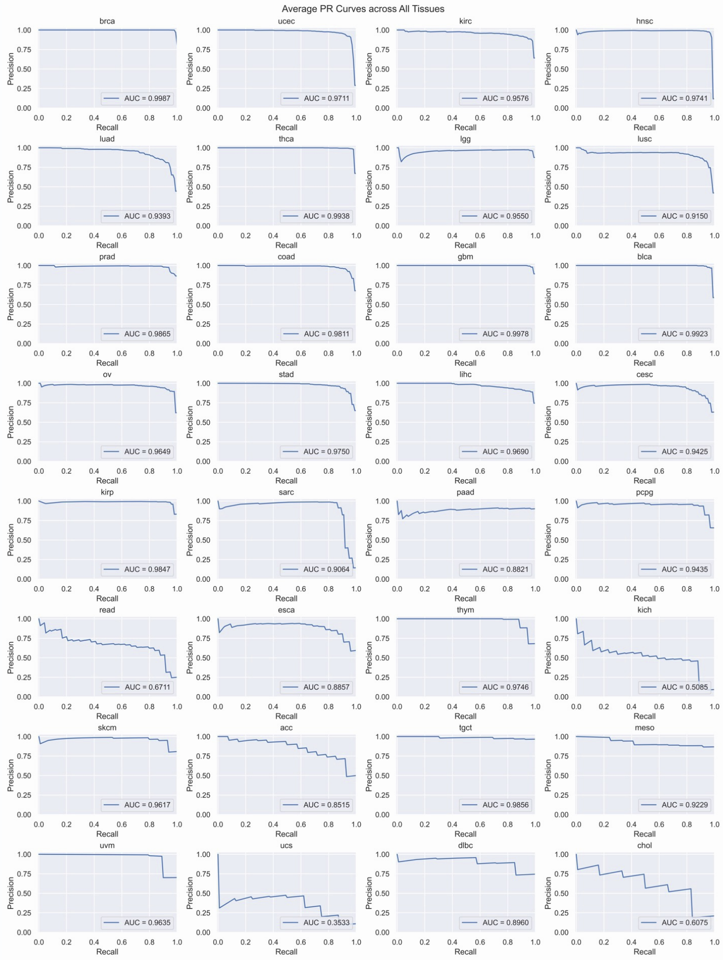

Figure S5. Related to Table 1. **Additional Characteristics of Final Dataset.** (A) Pages per Report, per-Tissue Distribution. (B) Tissue Sites (Institutions) per-Tissue Distribution. (C) Age Distribution. (D) Race Distribution. (E) Gender Distribution.

(A)

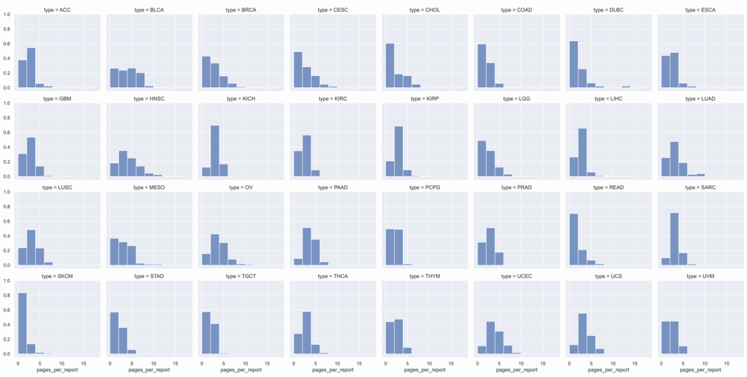

(B)

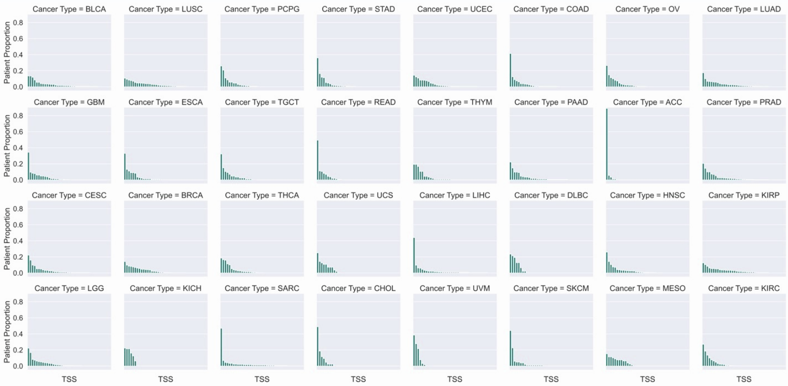

(C)

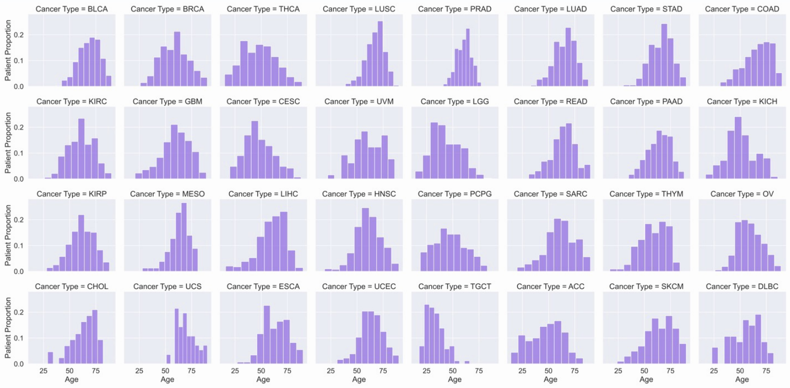

(D)

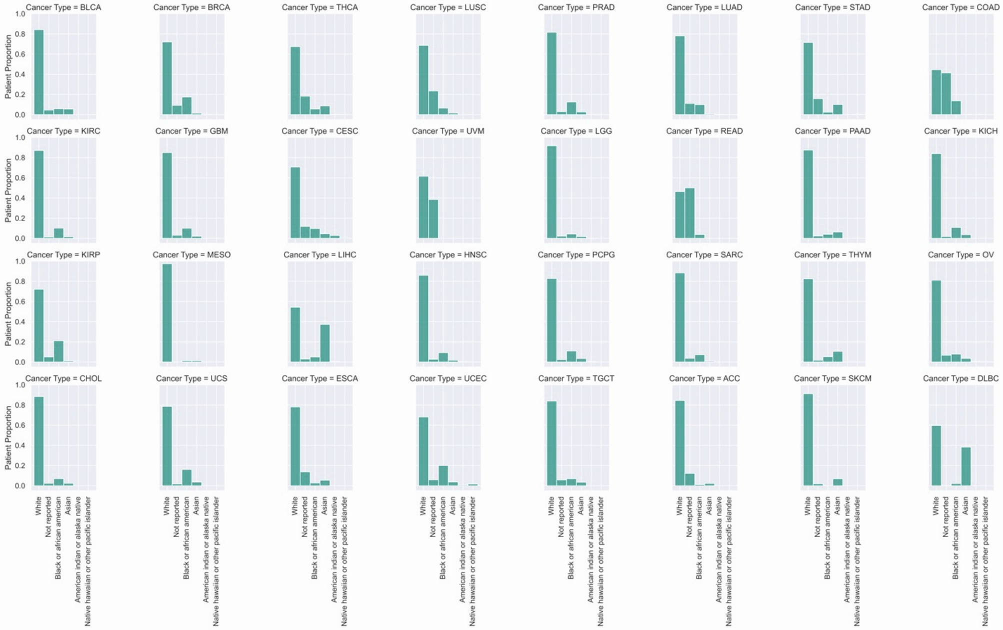

(E)

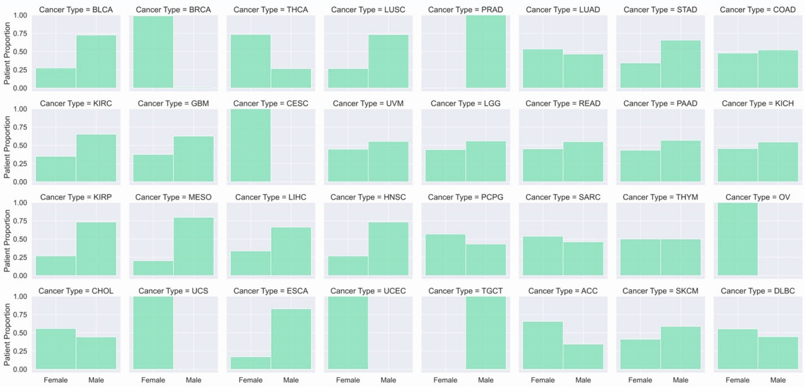

Table S1. Patients per cancer type in final dataset. Related to Figure 1.

Cancer Type	Number Patients Removed	Percent Patients Removed	Number Patients Remaining	Percent Patients Remaining
BRCA	63	5.74	1034	94.26
UCEC	2	0.36	546	99.64
KIRC	12	2.23	525	97.77
HNSC	8	1.52	520	98.48
LUAD	34	6.51	488	93.49
THCA	20	3.94	487	96.06
LGG	46	8.93	469	91.07
LUSC	36	7.14	468	92.86
PRAD	54	10.8	446	89.2
COAD	41	8.93	418	91.07
GBM	196	32.94	399	67.06
BLCA	33	8.01	379	91.99
OV	216	36.8	371	63.2
STAD	82	18.51	361	81.49
LIHC	36	9.55	341	90.45
CESC	18	5.86	289	94.14
KIRP	11	3.78	280	96.22
SARC	12	4.6	249	95.4
PAAD	9	4.86	176	95.14
PCPG	5	2.79	174	97.21
READ	8	4.71	162	95.29
ESCA	39	21.08	146	78.92
THYM	10	8.06	114	91.94
KICH	1	0.88	112	99.12
SKCM	368	78.3	102	21.7
ACC	2	2.17	90	97.83
TGCT	47	35.07	87	64.93
MESO	8	9.2	79	90.8
UVM	15	18.75	65	81.25
UCS	1	1.75	56	98.25
DLBC	1	2.08	47	97.92
CHOL	2	4.44	43	95.56

Table S2. Demographic table across train and test sets. Related to Table 1 and Methods ("Cancer Type Classification").

	N (Train)	P (Train)	N (Test)	P (Test)
**Age**				
0-18	12	0.1	1	0.1
18-29	236	2.9	43	3
30-39	540	6.7	91	6.4
40-49	1043	12.9	183	12.8
50-59	1881	23.2	349	24.4
60-69	2288	28.3	383	26.8
70-79	1575	19.5	275	19.2
80+	501	6.2	99	6.9
Not Reported	18	0.2	5	0.3
**Gender**				
Female	4243	52.4	792	55.4
Male	3851	47.6	637	44.6
**Ethnicity**				
Hispanic or Latino	282	3.5	61	4.3
Not Hispanic or Latino	5972	73.8	1023	71.6
Not Reported	1840	22.7	345	24.1
**Race**				
American Indian or Alaska Native	24	0.3	3	0.2
Asian	355	4.4	68	4.8
Black or African American	782	9.7	143	10
Native Hawaiian or Other Pacific Islander	12	0.1	1	0.1
Not Reported	771	9.5	162	11.3
White	6150	76	1052	73.6
